# Accelerating Recovery from Exercise-Induced Muscle Injuries in Triathletes: Considerations for Olympic Distance Races

**DOI:** 10.3390/sports7060143

**Published:** 2019-06-13

**Authors:** Thilo Hotfiel, Isabel Mayer, Moritz Huettel, Matthias Wilhelm Hoppe, Martin Engelhardt, Christoph Lutter, Klaus Pöttgen, Rafael Heiss, Tom Kastner, Casper Grim

**Affiliations:** 1 Department of Orthopedic, Trauma, Hand and Neuro Surgery, Klinikum Osnabrück GmbH, Osnabrück 49076, Germany; matthias.hoppe@klinikum-os.de (M.W.H.); martin.engelhardt@klinikum-os.de (M.E.); casper.grim@klinikum-os.de (C.G.); 2 Deutsche Triathlon Union (DTU), Frankfurt 60528, Germany; tom.kastner@iat.uni-leipzig.de; 3 Department of Orthopedic Surgery, Friedrich-Alexander-University Erlangen-Nuremberg, Erlangen 91054, Germany; isabel.mayer@gmx.de (I.M.); moritz.huettel@gmx.de (M.H.); 4 Department of Movement and Training Science, University of Wuppertal, Wuppertal 42119, Germany; 5 Department of Orthopedics, Rostock University Medical Center, Rostock 18057, Germany; christoph.lutter@googlemail.com; 6 Department of Sports Orthopedics, Sports Medicine, Sports Traumatology, Klinikum Bamberg, Bamberg 96049, Germany; 7 B·A·D Group, Darmstadt 64295, Germany; klaus@drpoettgen.de; 8 Department of Radiology, University Hospital Erlangen, Erlangen 91054, Germany; rafael.heiss@klinikum-os.de; 9 Department of Sport Medicine Humboldt University and Charité University Medicine, Berlin 10117, Germany; 10 Institute for Applied Training Science Leipzig (IAT), Leipzig 04109, Germany

**Keywords:** DOMS, EIMD, recovery, regeneration, muscle injuries, CWI, compression, endurance

## Abstract

The triathlon is one of the fastest developing sports in the world due to expanding participation and media attention. The fundamental change in Olympic triathlon races from a single to a multistart event is highly demanding in terms of recovery from and prevention of exercise-induced muscle injures. In elite and competitive sports, ultrastructural muscle injuries, including delayed onset muscle soreness (DOMS), are responsible for impaired muscle performance capacities. Prevention and treatment of these conditions have become key in regaining muscular performance levels and to guarantee performance and economy of motion in swimming, cycling and running. The aim of this review is to provide an overview of the current findings on the pathophysiology, as well as treatment and prevention of, these conditions in compliance with clinical implications for elite triathletes. In the context of DOMS, the majority of recovery interventions have focused on different protocols of compression, cold or heat therapy, active regeneration, nutritional interventions, or sleep. The authors agree that there is a compelling need for further studies, including high-quality randomized trials, to completely evaluate the effectiveness of existing therapeutic approaches, particularly in triathletes. The given recommendations must be updated and adjusted, as further evidence emerges.

## 1. Introduction

The triathlon is a multisport endurance sport in which athletes race sequentially in swimming, cycling, and running over various race distances. Due to an ever-expanding participation base and increased media interest, the triathlon is considered to be one of the fastest growing sports globally [1,2,3]. There are numerous event formats for both competitive and recreational events, ranging from super-sprint to long-distance triathlon, over which the International Triathlon Union (ITU) presides. In addition to the standard distance (Olympic distance) and sprint distance races, the ITU is the Governing Body overseeing the Mixed Team Relay (MTR) [4]; an innovative racing format where athletes complete a super-sprint triathlon, comprising of a 300 m swim, 6.6 km cycle and 1 km run before handing over to a teammate in the given order of female-male-female-male. The Mixed Relay is commonly held within 2–3 days of the individual race embedded within the same event. Having recently been included in the Olympic program, the Mixed Relay will make its debut at the Tokyo 2020 Olympic Games [4]. As well as being an exciting addition for spectators, it alters the demands placed upon the athletes, requiring them to work at higher intensity and greater speed throughout.

**Figure 1:**
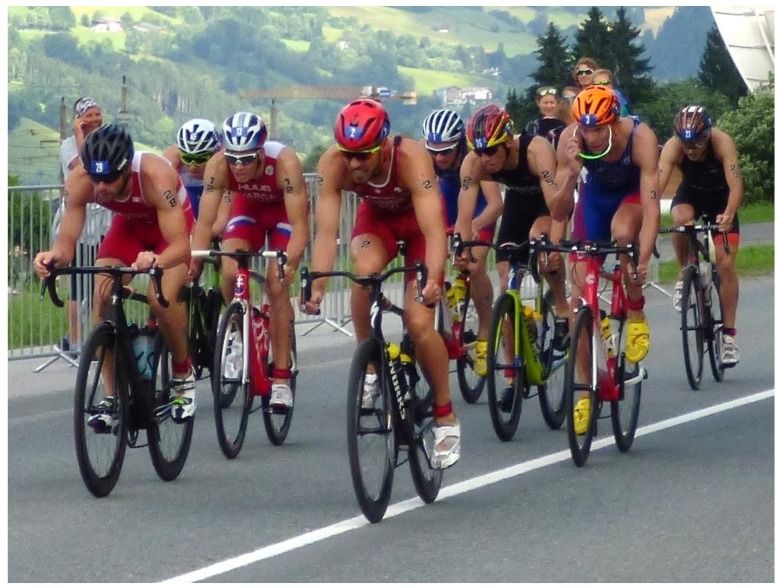
Scuffles for positions during cycling. High muscular demands are required. Elite triathletes need to perform i.e., over 1000 watts 3s peak power.

Historically, events such as the World Triathlon Series (WTS) or Olympic Games consisted of one intense and exhausting race for each athlete. Subsequently, athletes were able to recover in the days following competition until the resumption of full training or further competition; a second race start during the same competition was usually not performed. The fundamental change in triathlons from a single to multi-start event increases the demands placed upon the athlete in terms of recovery and prevention of exercise-induced muscle damages, which in turn has created a demand for research into optimizing recovery and regeneration. According to previous studies, particularly carbohydrate depletion, dehydration, hypoglycemia, electrolyte imbalance and ultrastructural muscle damage have been estimated to have a key function affecting muscle force output in triathletes [5,6]. As triathlon was established as a traditional endurance sport, common recovery practices focus mainly on the metabolic aspects (i.e., energy and carbohydrate storage, fluid intake, natrium balance [7,8]) and triathletes seem well informed about the necessity of rehydration after exercise [6]. Several studies demonstrated the presence of exercise-induced muscle injuries in triathletes [6,9,10], which is a relevant factor that impairs muscle function in triathlons, and which is known to mainly result from the running leg [6]. It was demonstrated in a recent study that post-exercise myoglobin and creatine kinase levels as indirect markers of muscle damage correlated with countermovement jumps height loss after the race [5,6]. The authors concluded that muscle fiber damage is one of the key factors for muscle fatigue in triathlons and strategies to lessen muscle fatigue during triathlon events should comprise a reduction in muscle damage [6]. However, those strategies have not been developed for triathletes up to now. Due to the adapted Olympic program, there is now a greater demand on athletes to regain high-intensity muscular activity and a high biomechanical output in order to deliver maximum performance for the MTR race [11] (Figure 1). 

Understanding the benefits of each intervention requires knowledge of the underlying muscle damaging and exhausting mechanisms, pathophysiological processes and treatment interventions [12]. The design of an “ideal” recovery regime is a topic receiving a lot of attention within current elite sports research [13,14,15,16]. Therefore, the aim of this narrative review is to provide an overview of the current literature on the pathophysiology as well as treatment and prevention of muscle soreness and suggesting the clinical implications for elite triathletes.

## 2. Materials and Methods

This narrative review focusses on the prevention and treatment of exercise-induced muscle injuries in relation to elite level triathletes. The existing literature on the pathogenesis, treatment, and prevention of exercise-induced muscle injuries, including original research reports, systematic and non-systematic reviews, book chapters and case reports was reviewed independently from their level of evidence. No systematic literature search was conducted. The aim of the present work was (1) to update the information about the current findings in pathogenesis of exercise-induced muscle injuries; (2) to apply current evidence for the treatment and prevention of exercise-induced muscle injuries in general; and (3) to discuss and evaluate the current findings by considering clinical and practical implications for triathlon races and competitions. For the purpose of this manuscript, the work done by an interdisciplinary authorship, consisting of biomechanists, sports scientists and sports physicians from the German Olympic Federation (DOSB) and the German Triathlon Union (DTU, Deutsche Triathlon Union), as well as scientists specializing in the field of exercise-induced muscle injuries is highlighted.

## 3. Results and Discussion 

### 3.1. Mechanisms and Pathogenesis of Exercise-Induced Muscle Injures 

#### 3.1.1. Differentiation between Muscle Soreness, Exercise-Induced Muscle Damage (EIMD) and Delayed Onset Muscle Soreness (DOMS)

**Figure 2:**
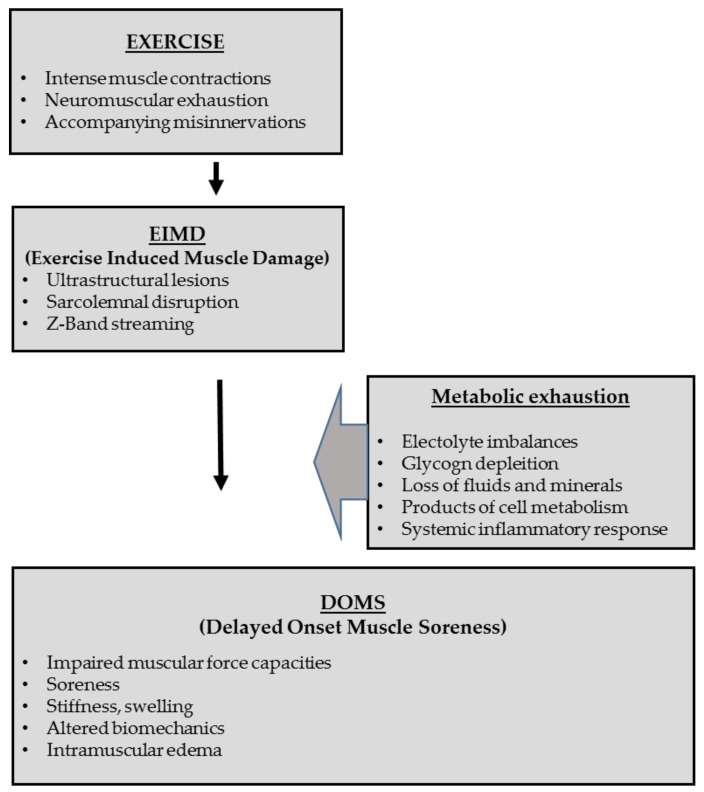
Illustrating the pathophysiological pathway of exercises, Exercise-induced Muscle Damage (EIMD), Delayed Onset Muscle Soreness (DOMS) and accompanying metabolic exhaustion; adapted from Heiss et al. [12].

**Figure 3:**
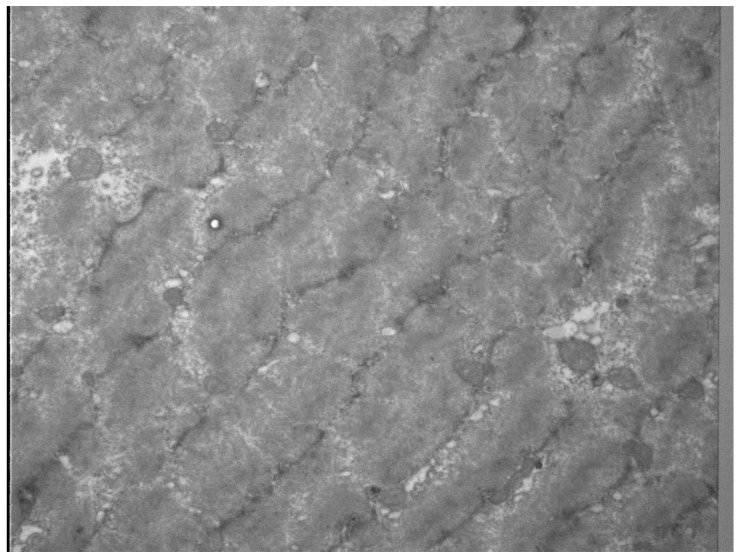
Z-disk disintegration and myofilament disarrangement as sign of ultrastructural damage was evaluated by electron microscopy of biopsies of human vastus lateralis 24 h after strenuous resistance exercise for 70 s under tension leading to DOMS (With kind permission, Prof. W. Bloch, German Sport University Cologne, Germany).

The clinical progression and manifestation of exercise-induced muscle damage, also known as DOMS, commonly begins 6–12 h post exercise, increasing progressively until peak pain occurs at 48–72hrs and thereafter decreasing until completely imperceptible 5–7 days post exercise [17,18]. DOMS is often accompanied by impaired muscle contraction and reduced force capacity [17,19,20], whilst a local or even global area of increased muscle tone is commonly observed [21,22,23,24]. DOMS is associated with local muscle soreness, reduced range of motion and altered biomechanical function of the adjacent joints [17,19,20,25,26]. Although the precise underlying causes of DOMS remain unknown [27], it is commonly accepted that the main mechanisms are related to ultrastructural damage of skeletal muscle integrity (exercise-induced muscle damage, EIMD) caused by intense and exhausting exercise and/or unfamiliar sporting activity [28,29,30] (Figure 2, Figure 3); for elite triathletes, the first scenario being the most relevant. 

In the past, eccentric contractions in particular were suspected to play a key role in the development of ultrastructural muscle injuries. In this condition, external loads are greater than the force generated by the muscles fibres under concentric conditions [12,28,31]. The higher muscular force is caused by an increase in recruitment of active cross-bridges [32] and in particular, by “passive-elastic” factors (i.e., Ca^2+^ triggered increased stiffness of titin and its winding on actin [12,33]), as described in the three-filament model and winding filament by Herzog et al. [34]. However, this hypothesis is not entirely relevant to triathletes, as eccentric contractions may occur solely during running, particularly on inclined surfaces. Several studies demonstrated the presence of exercise-induced muscle injuries in triathletes [6,9,10]. However, the exact cause of muscle damage in endurance sports, in particular in triathletes in triathletes has yet to be elucidated. In triathlons, there are no isolated eccentric contractions that induce a “pure eccentric overload” as applied in several DOMS models. Instead, the disciplines of swimming, cycling, and running are associated with changes of direction and positioning and eccentric contractions are shorter and part of the entire stretch-shortening cycle [35,36]. Swimming and cycling are considered to produce minor muscle damage in the involved muscles. Running however, as weight bearing activity includes concentric and eccentric actions in the lower extremity muscles [6]. During high-intensity races, central neuromuscular exhaustion and accompanying misinnervations can be discussed to reinforce the development of ultrastructural muscle injuries in these conditions. Hence, weariness induced alterations in intra- and intermuscular coordination between the muscle fibres with associated overstressing and damaging single muscle fibers should also be considered a factor [23]. Furthermore, it is unclear if metabolic exhaustion may also reinforce the development of DOMS. 

#### 3.1.2. Inflammatory and Healing Responses 

**Figure 4:**
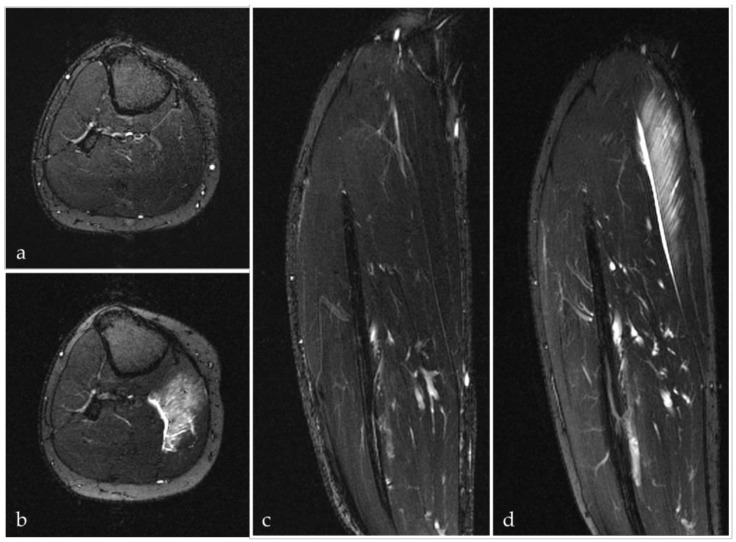
Axial (**a**,**b**) and coronal (**c**,**d**) T2-weighted fat-suppressed MRI images of the lower leg before (**a**,**c**) and 60 h after eccentric exercise (**b**,**d**) in the same participant. The increased signal intensity (**b**,**d**) reflects a rising fluid content in the gastrocnemius medialis muscle as equivalent of DOMS.

Electrolyte imbalances, leukocyte accumulation and infiltration in the exercised muscle, as well as an upregulation of circulating pro-inflammatory cytokines have been found in the context of DOMS as well as after exhausting endurance trials [27,28,29,37]. The released cytokines lead to a higher vascular permeability and microcirculation disturbances as they act as inflammatory mediators [38]. The presence of interstitial fluid associated with intramuscular edema and compartment swelling (Figure 4) as well as the presence of diverse inflammatory substances are described to be responsible for nociceptor activation and pain sensation [39,40]. In response to the loss of sarcolemmal integrity and permeability of the plasma membrane, DOMS is associated with increased creatine kinase (CK) activity levels, which are one of the leading indirect markers of muscle damage [19,38,41,42]. Several further markers such as Interleukin 6 (IL-6), C-reactive protein (CRP) [43], PTX-3 or LDH are upregulated during inflammation within damaged tissues [44]. However, the assessment of these parameters has been studied primarily in a scientific setting, as it is not methodological feasible in the context of triathlon competitions; therefore, generalizability is limited. 

## 4. Treatments and Strategies to Target Exercise-Induced Muscle Injuries

It is desirable to prevent athletes from experiencing DOMS, particularly in the context of multi-start events. Based on pathophysiological factors, recovery strategies should focus on various aspects, including (1), the primary prevention of ultrastructural lesions during exercise (prevention of EIMD), (2), the treatment of inflammatory responses leading to DOMS, and (3) – in case of failure of point (1) and (2) the treatment and recovery strategies of reduce signs of DOMS [12]. A range of interventions aiming to either prevent or relieve the symptoms of DOMS, thereby accelerating the recovery process from this performance-limiting condition, have been reported. 

### 4.1. The Role of Sleep

A reduction in total regeneration time at major sporting events containing multiple starts increases the awareness of sleep as a crucial component of the recovery process. Disturbance of sleep can be caused by early morning training, increases in training load, travel departure times, jet lag and altitude [45,46]. Sleep deprivation is associated with lower physical and mental performance [47], whilst optimal levels of sleep (in both quantity and quality) have been linked to increased performance and reduced risk of injury [48]. The greater the demand for speed, tactical awareness and technical skill within a sport, the more sensitive it is to sleep duration manipulation. Further to this, longer-term sleep manipulation is more likely to affect athletic performance than acute sleep manipulation [49]. Some studies showed a positive effect of sleep extension on subsequent performance [50,51]. A promising option in the case of an insufficient night’s sleep could be the introduction of power naps, provided that the recommendation of a 30-min maximum duration is not exceeded [52,53]. For practical suggestions, refer to Table 1.

### 4.2. Compression Therapy

In recent years, there has been increasing interest in the efficacy of compression garments for post-exercise treatment in various disciplines. Existing systematic reviews have concluded that compression garments are effective in enhancing recovery from exercise-induced muscle damage [55,56], whilst the majority of previous studies failed to support a performance-enhancing effect of wearing compression garments during exercise. Results are not uniformly accepted. Inconsistent findings may be caused by heterogeneous conditions [55,57] and no consensus for optimal application time, applied pressure, composition as well as shape and design [57] could be provided thus far. Overall there is limited evidence to suggest that compression garments impact athletic performance [58,59,60], but there may be legitimation for its use in terms of recovery [55,57,61,62]. 

#### 4.2.1. Compression Therapy during Exercise

Bringard et al. suggest that wearing compression garments during exercise may enhance muscle perfusion and decrease muscle oscillation which thereby promotes a lower energy expenditure and an improvement or maintenance in submaximal running speed [63]. Moreover, it is reported that compression garments lead to increased comfort [12,57,64] and support the underlying tissue resulting in reduced microtrauma and muscular damage [62,65]. In contrast, a systematic review by Beliard et al. reported that nine from ten studies did not show any performance enhancing effects [57], nor significant changes in objective physiological variables such as increased oxygen uptake or plasma level related changes of lactate or CK [12,63,64,66]. Only Kemmler et al. could demonstrate a significant performance effect (time under load, total work) for calf compression in male runners [66]. In summary, wearing compression garments during exercise (triathlon) might lead to marginal improvements in performance; however, evidence is inconclusive. 

#### 4.2.2. Compression Therapy Post Exercise

For post exercise compression therapy, heterogeneous modalities are discussed. For the prevention of DOMS, several studies have shown positive effects on recovery, function and strength of the treated muscle groups [12,55,57,67]. The decrease of CK levels shown in two studies [15,55,57] and the reduction of lactate level [67,68] could be seen as valid markers for improved regeneration. This could be attributed to an attenuation in the release of CK into the bloodstream, improved clearance from the circulation and enhanced repair of the damaged muscle tissue [12,57]. This theory could however not be confirmed by MRI based investigations which showed no reduction in edema or DOMS in the calf muscles [24]. In the same study, the only parameter shown to be significant was a faster normalization of muscle stiffness.

#### 4.2.3. Intermittent Compression Therapy

A further available treatment strategy is intermittent compression therapy. The NormaTec Pulse Massage Pattern (The NormaTec PULSE recovery Systems, NormaTec, Watertown, Massachusetts, USA) works with a pulsating, dynamic, upwards moving compression with the goal of mobilizing fluids and supporting the outflow out of the extremities [12]. This method is poorly investigated with only one study exists showing local hyperermia [69]. Pneumatic compression pants did not alter the rate of muscle glycogen resynthesis, blood lactate, or blood glucose and insulin concentrations associated with a post-exercise oral glucose load [70]. Further, Haun et al. reported a decrease in DOMS and potential reduction in muscle proteolysis as well as oxidative stress [71]. There is a need for further research related to mechanistic changes. 

Practical implications: Based on the evidence reviewed, there is no generalized recommendation for use of compression garments during exercise, but if the athlete reports subjective benefits, then it may have a positive impact. There is evidence suggesting that compression can play a role in enhanced post-exercise recovery, whilst evidence for dynamic compression is inconclusive. 

### 4.3. Thermal Therapy 

#### 4.3.1. Cold Water Immersion Therapy (CWI)

Systemic cooling for post-exercise recovery and regeneration has become more widespread in elite sport. Specifically, CWI has established itself as an effective therapy to cope with EIMD and alleviate physiological and functional deficits [72]. There is high level evidence for its clinical effectiveness in regard to an enhanced regeneration [12]. Hohenauer et al. emphasized, that cooling is superior in comparison to passive recovery strategies with CWI achieving the best results in reducing the symptoms of DOMS [73]. This was confirmed by Leeder et al., especially up to 96 hrs post exercise [74]. Various aspects promoting a faster regeneration are suggested, for example, a facilitated removal of metabolites [75]; limitation of inflammation and cell damage [76]; analgesic effects through activation of M8 cation channels (TRPM8), located in a and c fibres [77]; compression through increased hydrostatic pressure [75] and especially a decreased tissue temperature, blood flow and cardiovascular strain [74,76,78]. Referring to the water temperature, the best results were found to be between 11 and 15 °C for 11–15 min. [72]. Also, the efficacy of CWI seems to be depended of the previous type of exercise. CWI is thought to be most effective in attenuating EIMD induced by whole body prolonged endurance-based exercise [79]. Inflammatory processes may be limited through a coldness dependent down regulation of intramuscular metabolism [80]; however, associated adverse effects with regards to glycogen resynthesis or lactate metabolism cannot be assumed and have to be investigated further. 

Practical implications: Taking into account the different practical aspects, CWI seems to be an appropriate short-term recovery modality of exercise-induced muscle injuries in triathletes, especially within the new framework that includes mixed relay competition. Beside positive muscle injury related effects, CWI should be viewed critically in regard to glycogen and energy metabolism. The application of CWI should be adjusted to suit the environmental conditions such as climate, local equipment, conditions and, not least, the athlete’s individual preference in order to be most effective. 

#### 4.3.2. Heat Therapy

The application of heat therapy is still controversially discussed, and studies have to be regarded cautiously in the examined context. Study protocols vary from experimental designs in isolated mouse soleus muscle to gain information about glycogen and energy metabolism [81], to studies focusing mainly on clinical outcomes like maximal range of movement (ROM) [82] or voluntary contraction and muscle steadiness after sauna [83]. In regards to the acute inflammatory response leading to DOMS (between 48–72hrs), whole-body heat therapy has to be regarded critically [28]. In contrast, after the outlined peak, heat therapy can support soft tissue repair, tissue nutrition and circulation [84,85]. Studies show a positive effect in gaining muscle strength associated with hypertrophy (10 weeks heat therapy without strength training) through provoked gene expression for growth and differentiation [12,86] enhanced recovery after eccentric resistance training with an acceleration of angiogenic factors in human knee extensor muscle [87] and an acceleration of muscle contractility properties and decreased muscle steadiness after whole-body heat therapy by sauna [83]. In contrast Frier et al. emphasizes pre-exercise heat stress may inhibit increases in muscle mass, potentially caused by accumulation of heat shock proteins in lower limbs of rats [88]. 

Practical implications: For the application of heat therapy in triathlons, no general recommendation can be made at this point. Especially for acute injuries in the first inflammation phase, cooling is the preferred strategy. After the inflammatory response or recovery without muscle injury, heat therapy can support regeneration and improved tissue healing. Unfortunately, there are no available studies with an endurance-based protocol, specifically relating to long-term muscle load such as the duration of a triathlon event. 

### 4.4. Active Regeneration

Conventional strategies such as low intensity exercise and stretching have a long tradition in sports [89], whilst there has been a surge in innovative techniques recently which are yet to be fully investigated. Low intensity training is suggested after eccentric or high-intensity training sessions inducing DOMS, which is associated with muscle pain and tenderness [90], as well as compromised performance and swelling [28]. It has been proposed that the short-term alleviation of pain during exercise is due to the breakup of adhesions in the sore muscles, an increased removal of noxious waste products via an increased blood flow or an increased endorphin release [90]. There are two studies inducing DOMS with an eccentric exercise protocol in upper arm [91] and wrist extensor muscles [92] followed by 8–10 min ergometry; neither was unable to show any clinical advantage [90]. In contrast Hasson et al. reported a significant decrease in DOMS 48 hrs after a high velocity concentric isokinetic exercise of the knee extensors and flexors by performing a stepping exercise 24 hrs after the initial training [90,93]. In summary, study results related to recovery from DOMS are heterogenous, without any clear conclusion.

Furthermore, in regard to fatigue inducing exercise, Vanderthommen et al. were unable to show any superior effect in performance or pain of active compared to passive regeneration after isometric muscle contraction [94]. The same recovery modality of pedaling on a bicycle ergometer with a moderate load had however demonstrated an improvement in the recovery process [94]. These studies showed a superior blood lactate removal following active recovery [95,96] compared to passive recovery. In a randomized controlled trial from 2018 no positive effects of dynamic contract-relax stretching in either strength or in ROM or pain threshold could be confirmed [97]. Moreover, stretching after eccentric exercises failed to prove its efficacy [90], therefore it was concluded that there is insufficient data for stretching as a strategy to enhance recovery [98].A new form of active regeneration is foam rolling, as a method of self-myofascial release, which has become popular in recovery [12] with ubiquitous use through all performance levels. Several studies have examined the effect of post muscle damaging exercise foam rolling. All studies reported a significant reduction of pain, whilst two also showed objective benefits in sprint and jump performance and in ROM [17,99], which was, however, contradicted by the findings of Jay et al. [100]. As another clinical outcome Fleckenstein et al. observed a significant effect of foam rolling on neuromuscular exhaustion as maximal isometric voluntary force of the knee extensors and pain [101]. The only study observing the rate of blood lactate clearance was conducted after a 100 m water-rescue in life guards which was also found to be significant [102]. In conclusion, foam rolling seems to be effective in pain reduction, but further benefits have yet to be conclusively reported. The underlying physiological principles and potential risks also remain unclear [103,104].

Practical implications: As an active recovery strategy, low intensity training in form of 15 min of pedaling directly after exercise might have a recovery enhancing effect, however, there is little evidence of performance enhancement or objective support for muscle healing [12].

### 4.5. Nutrition

The importance of post-exercise nutrition as a critical component of recovery is widely accepted. However, evidence-based dietary recommendations specifically related to elite triathletes are still lacking. There are many studies evaluating the effects of nutritional expression of DOMS after EIMD. The importance of protein supplementation for endurance triathletes is increasingly accepted. Post-exercise protein supplementation enhances muscle protein synthesis and satellite cell activity for muscle repair, furthermore facilitates muscle glycogen resynthesis [105]. Beside this, branched-chain amino acids (BCAAs) which are mainly metabolized in skeletal muscle [106], are thought to have further positive effects on exercise-related cytokine production in cases of structural and metabolic processes due to exercise damage [12,107]. A review analyzing the effects of branched-chain amino acids (BCAAs) in endurance sports concluded that supplementation with BCAAs lowers the degree of pain and muscle damage, perceived exertion and mental fatigue, but stimulates the anabolic response in recovery and improves the immune response. There was no consensus about the dose and timing, but it seems to be most effective if there is 2–3/1 1g relationship between leucine/ isoleucine and valine amino acids [108]. Doering et al. suggest that masters athletes may have slower recovery rates due to impaired muscle remodeling mechanism, compared to younger, equally trained athletes, after muscle-damaging endurance exercise. Given this fact, masters athletes could benefit from higher doses of post exercise dietary protein intake, especially with leucine [105]. A systematic review showed that a high daily BCAA supplementation (>200 mg kg ^−1^ day ^−1^) for a long period (>10 days) was particularly effective when the extent of muscle damage was low-to-moderate and consumed pre-exercise [12,40,109]. Another important nutritional component targeting a fast and ideal recovery are the omega-3-fatty acids. By limitation of anti-inflammatory responses and oxidative stress, omega-3-fatty acids significantly reduce the DOMS sensations. Therefore, it is recommended to ingest 1.8–3 g of omega-3-fatty acids after exercise [110,111].

## 5. Conclusions

The present work provides an overview of the pathophysiological pathway, as well as the various treatment strategies in the field of exercise-induced muscle injuries, and evaluates their effectiveness with respect to the existing scientific evidence and practical expertise (Table 2). As a limitation of this review, there are only a few studies dealing with specific interventions in elite triathletes. Most existing investigations focusing on the pathogenesis or interventions in exercise-induced muscle injuries consist of a wide spectrum of athletes and different accompanying metabolic demands. Further, when interpreting triathlon specific data, different metabolic demands in short and long-distance athletes have to be considered, and it is unclear if given general recommendations can be completely transferred to Olympic triathletes. The authors agree that there is a compelling need for further studies, including high-quality randomized trials, to completely evaluate the effectiveness of existing therapeutic approaches. The given recommendations must be updated and adjusted as further evidence emerges. 

## Figures and Tables

**Table 1:** Recommendations for improving sleep in athletes (Data adapted from Simpson et al. [54]).

**Obtain adequate total sleep duration**	Strategy 1: Track sleep for 2 weeks using a self-report sleep diary. Gradually increase sleep duration by 15 min every few nights, until athlete feels well rested and alert during the day. Consider increasing nighttime sleep by 30–60 min/night; this is particularly important if average sleep duration is <7 h/night	Strategy 2: Consider implementing regular naps, beginning on weekends or off-days if needed. Allow adequate time to return to full alertness after daytime naps
**Maintain healthy sleep habits**	Strategy 1: Develop a good sleep environment: the ideal room is cool, dark, and comfortable. Avoid having/using electronics or personal devices in bedroom	Strategy 2: Avoid alerting factors in the evening. Reduce ambient light exposure in late evening hours if possible, limit electronic device use at least 1 h prior to bedtime, allow for a 30–60 min relaxing wind-down period before bed. Ideally, consume no caffeine after lunch; limit alcohol use in late evening
**Minimizing impact of travel**	Strategy 1: Factor-in time needed to adjust to new time zone; as a rule of thumb, the body can adjust to 1 hr of time zone difference each day. Consider starting to shift body clock prior to departure or during flight; personalized travel planners (available online) may be helpful	Strategy 2: Reduce impact of non-jet lag travel effects: dehydration, acoustic stress, low physical activity, changes in food/drinking patterns

**Table 2:** Overview of discussed interventions in treatment and prevention of exercise-induced muscle damages.

Intervention		Practical Implications
**Compression**	Compression therapy during exercise	Overall there is evidence to suggest that compression garments are effective in the treatment of exercise-induced muscle damages (i.e., 6 h use in post-exercise set-up) Inconsistent data exists in terms of accelerating performance No recommendations can be made in regard to pressure level or design. Athlete’s Individual preference and comfort should be considered
	Compression therapy post exercise	
	Intermittent compression therapy	
**Thermal therapy**	Cold water immersion therapy (CWI)	CWI seems to be an appropriate short-term recovery modality of exercise-induced muscle injuries in triathletes Besides positive muscle injury related effects, the effects of CWI on glycogen and energy metabolism should be considered critically. The application of CWI should be adjusted to suit the environmental conditions such as climate, local equipment, conditions and not least the athlete’s individual preference in order to be most effective.
	Whole body cryotherapy	
	Heat therapy	
**Active regeneration**	Low intensity exercise	As an active recovery strategy, low intensity training in form of 15 min of pedaling directly after exercise might have a recovery enhancing effect, however, there is little evidence of performance enhancement or objective support for muscle healing No clear recommendations can be made on stretching or foam rolling
	Stretching	
	Foam rolling	
**Oral medications and nutrition**	Protein supplementation, use of branched-chain amino acids (BCAAs)	Post-exercise protein supplementation enhances muscle protein synthesis and satellite cell activity for muscle repair, furthermore facilitates muscle glycogen resynthesis Branched-chain amino acids (BCAAs) are thought to have further positive effects on exercise-related cytokine production in cases of structural and metabolic processes due to exercise damage
**Improving sleep**	Adequate total sleep duration Healthy sleep habits Mimimizing impact on travel	Please refer to Table 1
